# SwinFishNet: A Swin Transformer-based approach for automatic fish species classification using transfer learning

**DOI:** 10.1371/journal.pone.0322711

**Published:** 2025-05-20

**Authors:** Ebru Ergün

**Affiliations:** Department of Electrical and Electronics Engineering, Faculty of Engineering and Architecture, Recep Tayyip Erdogan University, Rize, Turkey; Kafkas University: Kafkas Universitesi, TÜRKIYE

## Abstract

The fish market is a crucial industry for both domestic economies and the global seafood trade. Accurate fish species classification (FSC) plays a significant role in ensuring sustainability, improving food safety, and optimizing market efficiency. This study introduces automatic FSC using Swin Transformer (ST) through transfer learning (SwinFishNet), which proposes an innovative approach to FSC by leveraging the ST model, a cutting-edge architecture known for its exceptional performance in computer vision tasks. The ST’s unique ability to capture both local and global features through its hierarchical structure enhances its effectiveness in complex image classification tasks. The model utilizes three distinct datasets: the 12-class BD-Freshwater-Fish dataset, the 10-class SmallFishBD dataset, and the 20-class FishSpecies dataset, focusing on image processing-based classification. Images were preprocessed by resizing to 224 × 224 pixels, normalizing, and converting to tensor format for compatibility with deep learning models. Transfer learning was applied using the ST, which was fine-tuned on these datasets and optimized with the AdamW algorithm. The model’s performance was evaluated using classification accuracy (CA), F1-score, recall, precision, Matthews correlation coefficient, Cohen’s kappa and confusion matrix metrics. The results yielded promising CAs: 0.9847 for BD-Freshwater-Fish, 0.9964 for SmallFishBD, and 0.9932 for the FishSpecies dataset. These results underscore the potential of the SwinFishNet in automating FSC and demonstrate its significant contributions to improving sustainability, market efficiency, and food safety in the seafood industry. This work offers a novel methodology with broad applications in both commercial and research settings, advancing the role of artificial intelligence in the fish market.

## Introduction

The accurate classification of fish species is a critical task in ecological research, environmental management, and the seafood industry [1]. Reliable species identification is essential for assessing marine biodiversity, monitoring ecosystem health, and enforcing conservation policies. Furthermore, it plays a pivotal role in preventing the mislabeling of seafood products, ensuring food safety, and maintaining the economic stability of fisheries. Traditional fish classification methods, which rely on manual identification by experts, are often time-consuming, error-prone, and impractical for large-scale applications. As a result, the demand for automated and highly accurate fish classification systems has grown significantly [2].

Recent advancements in artificial intelligence, particularly in deep learning (DL) and computer vision, have revolutionized image-based classification tasks. Among these, transformer-based architectures have demonstrated remarkable performance in extracting both local and global features from images, enabling superior classification accuracy (CA). The Swin Transformer (ST), an advanced vision transformer (ViT), is particularly well-suited for complex image recognition tasks due to its hierarchical feature extraction mechanism. Unlike conventional convolutional neural networks (CNNs), the ST effectively captures multi-scale spatial dependencies while maintaining computational efficiency [3]. This characteristic makes it an ideal choice for fish species classification (FSC), where subtle differences in texture, shape, and color are critical for accurate identification.

In this study, we proposed an approach for automatic FSC using the ST with transfer learning (SwinFishNet). To evaluate the effectiveness of our method, we employed three diverse datasets: the 12-class BD-Freshwater-Fish dataset, the 10-class SmallFishBD dataset, and the 20-class FishSpecies dataset. The images in these datasets undergo preprocessing steps, including resizing to 224×224 pixels, normalization, and tensor conversion to ensure compatibility with DL frameworks. We leveraged the pre-trained ST model, fine-tuning it on these datasets while optimizing its performance using the AdamW algorithm. The main contributions of this study are as follows:

- The SwinFishNet is introduced for FSC, leveraging its hierarchical attention mechanism to enhance feature extraction and improve CA.
- Extensive experiments are conducted on three benchmark datasets, demonstrating the robustness and generalizability of the proposed approach.
- To ensure optimal adaptation to FSC tasks, the ST is fine-tuned using transfer learning while maintaining computational efficiency.
- Model performance is evaluated using CA, precision (PR), recall (RC), F1-score (F1), Matthews correlation coefficient (MCC), Cohen’s kappa (κ), confusion matrices and area under the curve (AUC), offering a comprehensive assessment of classification effectiveness.

## Associated works

In the domain of computer vision, image classification has emerged as a central research area, particularly for applications related to FSC [4]. Traditional machine learning techniques often require manual feature extraction, a process that can be time-consuming and might lack generalizability across varied datasets. However, with the rapid advancement of DL, driven by increased computational resources and more refined algorithms, significant progress has been made [5]. In particular, CNNs have played a key role in automating both feature extraction and classification processes, leading to remarkable advancements in fields such as fruit and vegetable classification. These developments have extended to various applications, including sorting, grading, variety identification, and disease detection, offering high accuracy and efficiency [4].

In the specific context of FSC, a number of studies have leveraged DL methodologies, with a predominant reliance on CNN-based models. Knausgard et al. proposed a two-step approach that utilized the YOLO object detection technique followed by a CNN with a Squeeze-and-Excitation architecture for classifying temperate fish species. This approach achieved a pre-training CA of 99.27%, and post-training CA of 83.68%, demonstrating both the model’s potential with large datasets and the importance of combining object detection with DL for improved classification results [6]. Building on YOLO-based object detection, Malik et al. developed FD_Net, a DL framework designed to identify multi-class fish species from camera images. This framework enhanced the standard YOLO approach by replacing Darknet53 with MobileNetv3, incorporating depthwise separable convolutions, and utilizing a Bottleneck Attention Module to improve feature extraction. Additionally, the model was optimized using DenseNet-169 and Arcface Loss, which contributed to better feature learning and an expanded receptive field. FD_Net demonstrated a 14.29% improvement in mean average precision over existing models such as YOLOv3, YOLOv4, YOLOv5, Faster-RCNN, and YOLOv7 [7]. Ahmed et al. proposed an embedded system that combined DL and the Internet of Things for FSC. Their model used two datasets featuring original and unsharp masked images of eight Bangladeshi fish species, which were tested with seven pre-trained ImageNet models. The best results, with an CA of 96.00%, were achieved by DenseNet121, DenseNet169, and DenseNet201, while a hybrid CNN+Convolutional LSTM model achieved an even CA of 97.00%, demonstrating the versatility of hybrid models in addressing classification challenges [8]. In another study, Qu et al. presented ConvFishNet, a model designed for FSC that utilized large convolutional kernels and depthwise separable convolutions to reduce model parameters. With its lightweight design and incorporation of PixelShuffle for improved upsampling, ConvFishNet achieved 88.44% PR on the WildFish dataset and 99.80% on the Fish4knowledge dataset, outperforming the FishNet model and setting a new standard for model efficiency [9]. Gao et al. introduced an optimized ResNet50 model for marine FSC. By augmenting a dataset of 30 species with additional preprocessing steps, they enhanced the model’s performance by incorporating a Dual Multi-Scale Attention Network and dropout regularization. Their optimized model achieved a 98.75% recognition CA, 3.05% higher than the standard ResNet50, and demonstrated generalization capabilities on both the ImageNet and QUT Fish Datasets, reaching CA of 97.65% and 98.75%, respectively [10]. Liawatimena et al. proposed a FSC system tailored for mobile devices, combining the YOLOv3 model with ResNet18 as the backbone. The dataset, consisting of 4000 images from local markets and online sources, featured four fish species. The model’s performance was benchmarked against SSD-VGG and a Huawei ExeML autogenerated model. The YOLOv3-ResNet18 model achieved an CA of 98.45% during training and 98.15% during evaluation [11]. Ayyad et. al developed a DL system for recognizing nine different types of fish, including Red Mullet, Sea Bass, Striped Red Mullet, and Shrimp. Using a dataset of 9000 images, the dataset was divided into training, validation and testing sets. Their model achieved outstanding results, with CA reaching 99.68%, PR at 99.69%, RC at 99.68%, and an F1 of 99.68% [12]. In another study, Veiga et al. addressed the challenge of fine-grained visual classification (FGVC) in fish species by combining the FGVC Plug-in Module (FGVC-PIM) with the ST. The FGVC-PIM focuses on the most discriminative image regions, while the ST provides robust hierarchical feature extraction. Tested across 14 datasets with 19 subsets under varying conditions, the method achieved results in 13 subsets, set new baselines in 2, and performed above 83.00% in the remaining 4 [13]. Recent advancements in FSC have been shaped by the foundational contributions of earlier research, paving the way for the integration of modern machine learning (ML) and DL techniques. While early studies are relied on simpler methods and limited datasets, contemporary approaches use large datasets and sophisticated modelling techniques to improve CA. Table 1 presents a comprehensive comparison of existing FSC methods, highlighting key aspects such as dataset characteristics, model selection and CA. This comparative analysis highlighted a significant shift from traditional techniques to DL-based models. In particular, CNN and advanced DL architectures showed significant improvements in classification performance, establishing themselves as the most effective approaches in the field. The table also illustrates the impact of dataset size and diversity on model performance. Studies using large datasets, such as the LifeCLEF14 dataset used by Mathur et al. [21] and the Fish4Knowledge dataset used by Chuang et al. [15], achieved higher accuracy rates. In contrast, studies using smaller datasets may face generalization challenges. Nevertheless, careful model selection and effective feature extraction techniques can enable strong classification performance even in scenarios with limited data availability.

**Table 1:** Recent advances in the classification of diverse fish species using state-of-the-art ML and DL.

References	Procedure						Result
	Dataset	Data Augmentation	Contribution	Number of Classes	Number of Images	Model	CA(%)
Fouad et al. [14]	Nile Tilapia Fish	No Augmentation	Automatic Nile Tilapia Fish Classification	2	151	Support Vector Machine (SVM) Algorithm	94.40
Chuang et al. [15]	Fish4Knowledge	Geometric Compatibility	Fish Classification	15	26418	Random Forest	93.80
Cueto et al. [16]	Koi Fish Species Image	No Augmentation	Koi Fish Classification	15	1500	CNN	84.00
Kartika et al. [17]	Koi Fish Species Image	No Augmentation	Koi Fish Classification	9	281	SVM	94.00
Alsmadi et al. [18]	Fish Image	No Augmentation	Fish Classification	24	500	Genetic Algorithm with a Back-propagation algorithm	87.00
Jose et al. [19]	Tuna Fish Image	Rotation, Shear, Zoom, Horizontal Flip, Brightness Adjustments, and Width-Height Translation	Tuna classification	3	612	CNN	93.91
Chhabra et al. [20]	Fish Image	No Augmentation	Automatic Fish Classification	8	435	VggNet16	93.80
Mathur et al. [21]	LifeCLEF14 fish	No Augmentation	Automatic Fish Classification	23	27370	ResNet50	98.44
Khotimah et al. [22]	Tuna Fish Image	No Augmentation	Tuna fish classification	8	60	Decision Tree Algorithm	88.00
Rathi et al. [23]	Fish4Knowledge	No Augmentation	Underwater FSC	23	27142	CNN	96.29
Islam et al. [24]	Indigenous Fish Image	No Augmentation	Indigenous Fish Classification	8	2610	SVM	90.00
Taheri-Garavand et al. [25]	Common Carp Fish Image	Rotation, Height Shift, Width Shift, Zoom, Horizontal Flip and Shear Intensity	Classifying Fish Freshness	4	2464	CNN	98.21

Unlike traditional CNN-based models, which mainly focus on local feature extraction, transformer-based architectures introduce a new paradigm in visual recognition by effectively modeling long-range dependencies. The ST, proposed by Liu et al., utilizes a hierarchical visual transformer structure supported by shifted window mechanisms, offering a balance between computational efficiency and feature representation power [26]. This design enables the model to capture both fine-grained local patterns and broader global structures, while maintaining linear computational complexity with respect to image size [26]. While ST has demonstrated success in tasks such as object detection and semantic segmentation, its application in FSC remains limited, highlighting a significant gap in the literature. Building upon these findings, this study introduces an innovative approach, SwinFishNet, which leverages the ST model with transfer learning for automatic FSC. The proposed approach aims to address challenges often encountered by traditional CNN-based methods, such as intra-class variability and interference from complex backgrounds. By fine-tuning the SwinFishNet model on three distinct datasets—the 12-class BD-Freshwater-Fish dataset, the 10-class SmallFishBD dataset, and the 20-class FishSpecies dataset—this approach seeks to improve CA. Thanks to the hierarchical attention mechanism of ST, the model highlights discriminative features between visually similar species, while offering strong generalization across different dataset conditions.

Previous studies have demonstrated the effectiveness of ST-based models in various classification tasks. For instance, Zhang et al. applied an ST-based model for mosquito species identification, achieving a CA of 98.20%, underscoring ST’s potential in species classification [27]. Similarly, Zu et al. developed the SwinT-SRNet framework for pollen image classification, overcoming challenges such as low resolution and complex backgrounds. Their model achieved CAs of 99.46% and 98.98% on the POLLEN8BJ and POLLEN20L-det datasets, respectively [28]. Furthermore, Shi et al. proposed STF-YOLO for small tea bud detection, combining ST and YOLOv8 networks. This model outperformed other detectors, achieving a CA of 91.50% and a mean average precision of 89.40% [29].

These findings strongly support the successful application of transformer-based models, like ST, across a wide range of classification tasks. However, the application of ST in FSC remains underexplored. By applying the ST model to FSC, we aim to fill this gap in the literature and improve the accuracy of FSC. SwinFishNet adapts ST’s advanced feature extraction capabilities to refine the classification process, offering a novel approach that distinguishes it from both traditional CNN-based and transformer-based FSC methods. This approach also lays a solid foundation for future research in this field. This study significantly contributes to the development of automated, accurate, and efficient FSC methods, advancing the field.

## Materials and methods

### Comprehensive description of the fish species datasets

#### BD-freshwater-fish dataset.

**Fig 1:**
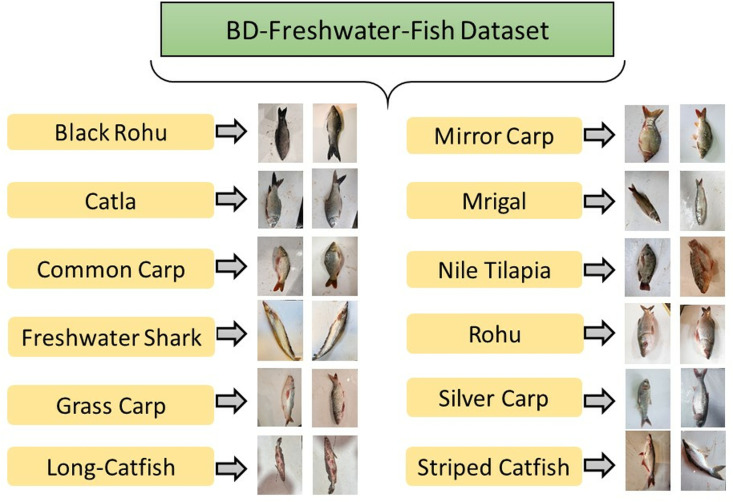
Structural representation of the BD-Freshwater-Fish dataset, illustrating its folder hierarchy and exemplar images for each fish category [ 30].

The BD-Freshwater-Fish Dataset serves as a comprehensive image repository designed to facilitate automated FSC using DL and computer vision techniques [30,31]. Given the complexity of visually distinguishing fish species due to variations in body morphology, fin structure, scale patterns, and other defining characteristics, this dataset is a valuable contribution to the domain of smart aquaculture and fisheries research. The dataset comprises 4389 high-resolution images, encompassing 12 distinct freshwater fish species indigenous to Bangladesh. The images were meticulously collected in natural market settings from the Sylhet and Jessore districts, ensuring a diverse representation of fish appearances. Each species in the dataset is categorized based on its scientific taxonomy, including Rohu, Catla, Mrigal, Grass Carp, Common Carp, Mirror Carp, Black Rohu, Silver Carp, Striped Catfish, Nile Tilapia, Long-Whiskered Catfish, and Freshwater Shark. Das et. al acquired the images using high-definition mobile cameras under natural lighting conditions, capturing both live and deceased fish specimens to maintain realism in classification tasks. Each fish was photographed out of water, presenting a lateral view to highlight distinguishing anatomical features. Representative images of each fish species are presented in Fig 1, providing visual insights into the dataset composition, while Table 2 details the distribution of images across different classes in the BD-Freshwater-Fish Dataset.

**Table 2:** Distribution of images in the BD-Freshwater-Fish Dataset by class.

Class Name	BD-Freshwater-Fish Dataset
	Number of images
Striped Catfish	460
Silver Carp	400
Rohu	514
Nile Tilapia	409
Mrigal	405
Mirror Carp	415
Long-whiskered Catfish	61
Grass Carp	410
Freshwater Shark	60
Common Carp	517
Katla	432
Black Rohu	306
Total	4389

### SmallFishBD dataset

**Fig 2:**
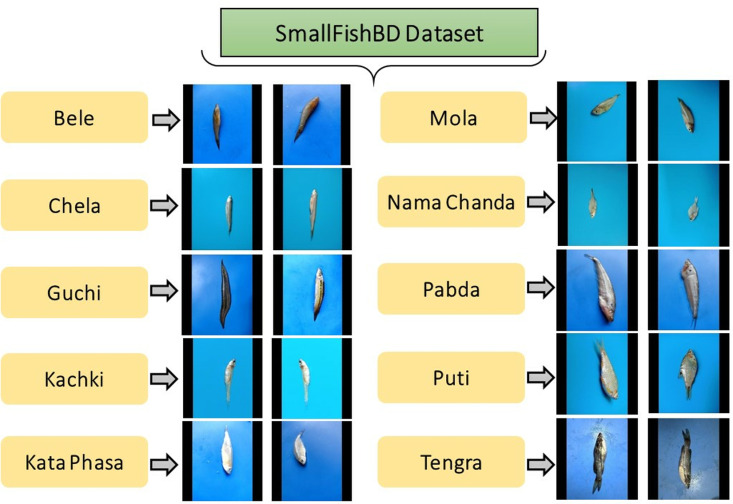
Systematic depiction of the SmallFishBD dataset, highlighting its organizational framework along with representative images corresponding to different fish species [ 32].

Ferdaus et al. meticulously curated the SmallFishBD Dataset, comprising high-resolution images of ten distinct small fish species commonly found in Bangladesh [32]. The dataset includes 1700 original images captured using high-definition smartphone cameras from various angles at local wholesale fish markets in Dhaka. To enhance model generalization, an augmented version was generated, expanding the dataset to 20400 images through transformations such as rotation, scaling, and flipping. The fish species represented in the dataset include Bele, Nama Chanda, Chela, Guchi, Kachki, Mola, Kata Phasa, Pabda, Puti, and Tengra, with each category stored in separate folders for systematic organization. The images are available in JPEG format with a standardized resolution of 320×320 pixels, ensuring uniformity across all samples. Fig 2 presents representative images from each fish category, while Table 3 provides the class-wise distribution of images in the SmallFishBD Dataset.

**Table 3:** Class-wise distribution of images in the SmallFishBD Dataset.

Class Name	SmallFishBD	
	Number of original images	Number of augmented images
Bele	205	2460
Nama Chanda	110	1320
Chela	190	2280
Guchi	164	1968
Kachki	247	2964
Mola	179	2148
Kata Phasa	129	1548
Pabda	125	1500
Puti	218	2616
Tengra	133	1596
Total	1700	20400

### FishSpecies dataset

**Fig 3:**
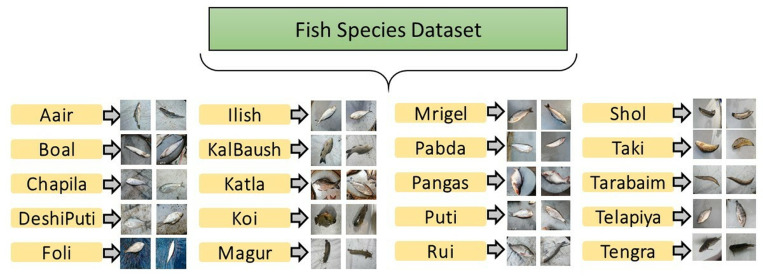
Hierarchical layout of the FishSpecies dataset, demonstrating its classification structure and sample images for various fish types [ 33].

The FishSpecies Dataset is a comprehensive collection of high-resolution images depicting a diverse range of fish species commonly found in Bangladesh by Sunny et. al [33]. These images were meticulously gathered from local fish markets, ensuring a realistic representation of natural variations in appearance, texture, and coloration across different species. Each image was manually categorized into one of 20 distinct classes, corresponding to specific fish species. This dataset preserves its raw form, maintaining the authenticity of real-world conditions. A total of 26950 images are systematically organized into 20 species-specific folders, providing a well-structured dataset for classification tasks. The dataset spans a variety of fish species, including commercially significant ones such as Hilsa (Ilish), Rohu (Rui), Catla (Katla), and Walking Catfish (Magur), among others. The number of images per species varies, reflecting the natural availability of these fish in the markets at the time of data collection. This class imbalance introduces an additional layer of complexity, making the dataset well-suited for evaluating the robustness of classification algorithms. To offer a clearer understanding, Table 4 provides a detailed breakdown of the image distribution across species, while Fig 3 presents representative samples from each category. These visual references highlight the dataset’s diversity, enabling researchers to assess intra-class and inter-class variations effectively.

**Table 4:** Class-wise allocation of images in the FishSpecies Dataset.

Fish Species Dataset			
Class Name	Number of images	Class Name	Number of images
Aair	1804	Mrigel	1808
Boal	1651	Pabda	1764
Chapila	428	Pangas	934
DeshiPuti	412	Puti	1560
Foli	562	Rui	2500
Ilish	1031	Shol	1424
KalBaush	917	Taki	2223
Katla	1765	Tarabaim	1262
Koi	842	Telapiya	2058
Magur	574	Tengra	1431
Total	26950		

### SwinFishNet-based methodology framework

In this study, a rigorous and well-structured methodology was devised to classify fish species using three high-resolution image datasets: the 12-class BD-Freshwater-Fish dataset, the 10-class SmallFishBD dataset, and the 20-class FishSpecies dataset. The primary objective was to develop a robust and accurate classification framework capable of effectively distinguishing fish species across these datasets by leveraging state-of-the-art DL techniques.

**Fig 4:**
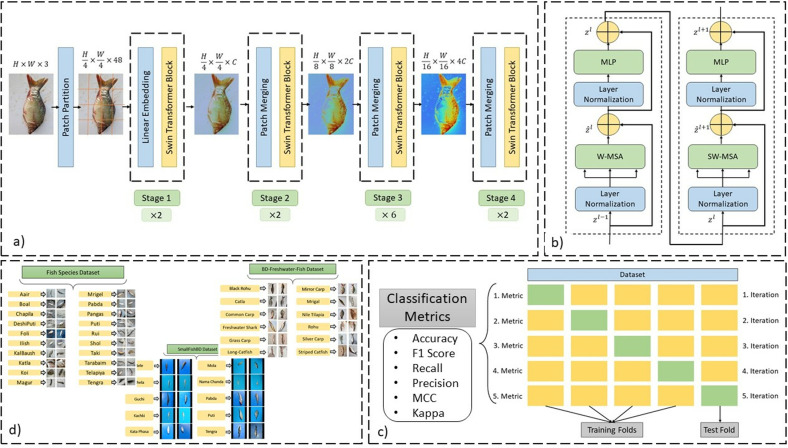
The optimized workflow of the proposed SwinFishNet methodology for high-precision FSC across diverse image datasets: **(a)** ST architecture, **(b)** ST blocks, **(c)** Classification procedure, and **(d)** Class distribution of each image dataset.

As illustrated in Fig 4, the proposed SwinFishNet methodology comprises a series of systematically designed steps to ensure precision, consistency, and reproducibility. The datasets were meticulously curated and preprocessed to enhance image quality and uniformity. All images were resized to a fixed resolution of 224×224 pixels and normalized using mean and standard deviation values extracted from the ST’s feature extractor. This preprocessing step was essential to align the input data with the model’s requirements, thereby improving classification performance across diverse datasets. To enhance model generalizability and robustness, a 5-fold cross-validation (5-FCV) approach was employed for each dataset. This ensured that the model was evaluated on multiple training and validation splits, minimizing potential biases and improving reliability. At the core of the methodology lies the ST, an advanced DL architecture recognized for its superior performance in image classification tasks. The model was fine-tuned to accommodate the specific characteristics of each dataset, with the classifier layer modified to match the corresponding number of output classes. Training was conducted over three epochs, employing the AdamW optimizer and CrossEntropyLoss function to facilitate stable convergence and optimal performance.

The model’s performance was rigorously assessed using a comprehensive set of evaluation metrics, including CA, PR, RC, F1, MCC, κ, and the AUC. These metrics were computed for each fold, and the results were aggregated to provide an in-depth evaluation of the model’s effectiveness. The software tools, libraries, and frameworks used in this study are detailed thoroughly. The experimental process was carried out using the Python programming language. For model training and fine-tuning, the PyTorch library, widely used in DL, was employed. Scikit-learn was used for statistical analysis, providing various tools for model evaluation and data manipulation. Matplotlib was utilized for visualization tasks, especially for plotting confusion matrices and performance graphs. The development environment chosen was PyCharm, an IDE optimized for Python programming, which facilitated efficient code management and debugging processes. The experimental setup was implemented on a machine equipped with an Intel(R) Core(TM) i7-9700K CPU running at 3.60 GHz and 8 GB RAM.

### Swin transformer

**Fig 5:**
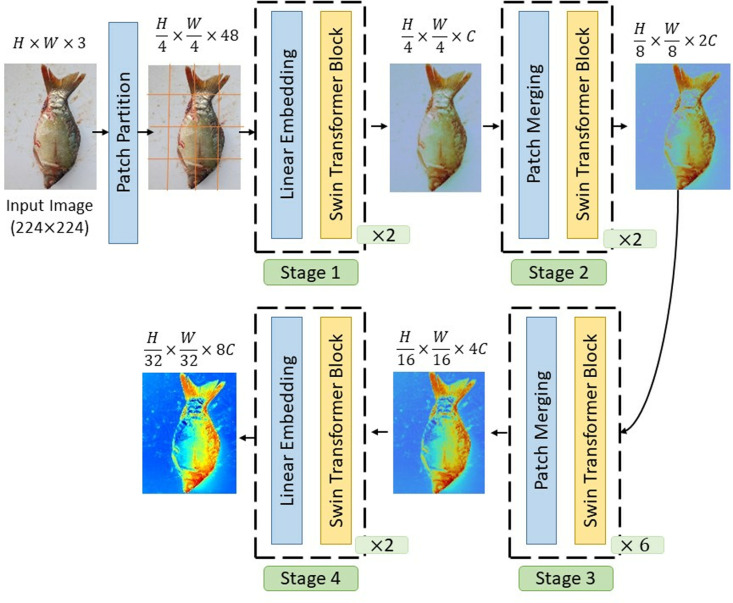
A comprehensive overview of the ST architecture.

**Fig 6:**
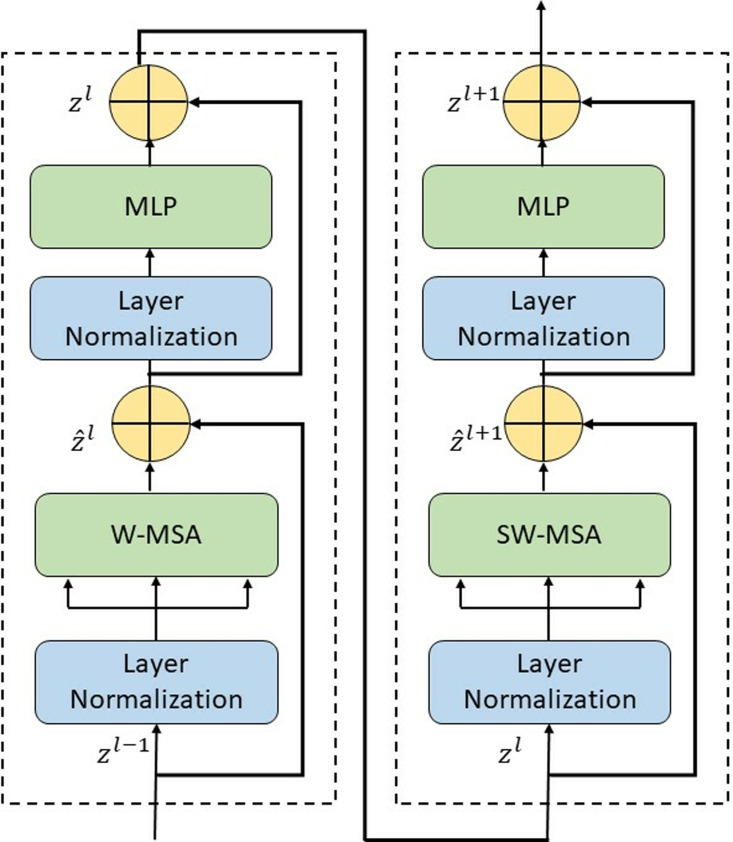
Structural representation of ST blocks.

Recent advancements in vision models have demonstrated the effectiveness of self-attention mechanisms in capturing both local and global dependencies. While conventional ViTs utilize a global attention mechanism, their quadratic computational complexity poses significant challenges, particularly for high-resolution inputs. To address this issue, ST introduces an efficient hierarchical framework with shifted window-based self-attention, optimizing computational efficiency while preserving crucial spatial relationships [34]. The hierarchical structure and multi-stage feature extraction approach of ST facilitate robust representation learning, as illustrated in Figs 5 and 6.

Unlike standard ViTs, which compute self-attention across the entire feature map, ST divides the input into non-overlapping windows, ensuring that self-attention operates within localized regions [34,35]. Given an input feature tensor X with dimensions H×W×C, where H and W denote spatial dimensions and C represents the number of channels, the attention scores within a window of size M×M are computed as in Equation 1.


$$\begin{array}{c} a_{ij}=\frac{\text{exp}(\frac{q_{i}\cdot k_{j}}{\sqrt{d}}+B_{ij})}{\sum_{j=1}^{M^{2}}\text{exp}(\frac{q_{i}\cdot k_{j}}{\sqrt{d}}+B_{ij})} \end{array}$$ (1)

where $a_{ij}$, represents the normalized attention weight between position i and j. $q_{i}$ and $k_{j}$ denote the query and key vectors, respectively. d is the feature dimension, influencing the scaling factor. $B_{ij}\;$accounts for the relative positional bias, enabling the model to incorporate spatial information. $M^{2}$, defines the number of elements in a window [35]. After computing the attention weights, they are applied to the corresponding value vectors $V_{j}$, yielding the refined feature representation as in Equation 2.


$$Z_{i}=\sum_{j=1}^{M^{2}}a_{ij}V_{j}$$ (2)

where $Z_{i}$ represents the updated feature at position i, obtained by aggregating contributions from all positions j within the same window. This localized approach significantly reduces computational complexity compared to global self-attention mechanisms.

However, restricting attention computation within independent windows limits information exchange across regions. To mitigate this, ST employs a shifted windowing mechanism, where windows in alternating layers are shifted by $\frac{M}{2}$ pixels. This shift facilitates cross-window interaction, ensuring that features from neighboring windows influence each other, thereby enhancing contextual representation. Unlike conventional ViTs, which maintain a fixed feature resolution throughout the network, ST progressively reduces spatial dimensions through patch merging, enabling multi-scale representation learning. Given a feature tensor $X^{\left(l\right)}$ at layer l with dimensions $H_{l}\times W_{l}\times C_{l}$, the transition to the next layer $X^{\left(l+1\right)}$ is performed by merging neighboring 2×2 regions and applying a linear transformation as given in Equation 3.


$$X^{\left(l+1\right)}=W_{m}\;(X_{1}^{\left(l\right)},\;X_{2}^{\left(l\right)},\;X_{3}^{\left(l\right)},\;X_{4}^{\left(l\right)})$$ (3)

where, $X_{1}^{\left(l\right)},\;X_{2}^{\left(l\right)},\;X_{3}^{\left(l\right)},\;X_{4}^{\left(l\right)}$ correspond to four adjacent feature vectors from the previous layer. $W_{m}$ is a learnable weight matrix that projects the concatenated features into a new representation with an increased channel dimension.

This hierarchical structure enables ST to efficiently model long-range dependencies while maintaining a computationally feasible framework. The ST blocks, responsible for feature extraction at each stage, are depicted in Fig 6, illustrating their internal components and the interaction between attention layers and feed-forward modules.

One of the key strengths of ST lies in its ability to balance computational efficiency and representational power. By restricting self-attention to local windows, the computational complexity per layer is reduced from $O(N^{2})$, as seen in standard ViTs, to O(N), making the model scalable to high-resolution images—an essential requirement for tasks such as object detection and segmentation. Furthermore, the hierarchical structure facilitates multi-scale feature extraction, enhancing the model’s ability to capture both fine-grained details and global contextual information.

Furthermore, Algorithm 1 comprehensively outlines the ST framework and the procedural steps followed in this study. It provides a detailed breakdown of the image classification pipeline, including data preprocessing, patch partitioning, hierarchical feature extraction, and model training. Additionally, it describes the optimization strategy, evaluation metrics, and cross-validation approach, ensuring a systematic and structured implementation of the proposed methodology.

**Algorithm 1. Swin Transformer**

 1: Input: $x\in R^{A\times T\times C}$, where A, T, and C denote the height, width, and number of channels of the input image tensor, respectively.

 2: Dataset Preparation: Define the dataset path and load images using the ImageFolder function.

 3: Patch Partitioning:

  •Compute the number of patches using N=(A/B)×(T/B), where B is the patch size.

  •Set B=4, resulting in patches of size 4 × 4.

 4: Feature Encoding: Encode patches using the Patch Partitioning and Linear Embedding mechanisms of the ST.

 5: Swin-Tiny Model Processing: The Swin-Tiny model consists of four hierarchical stages with transformer block configurations of (2, 2, 6, 2).

  •For each transformer layer i from 1 to $N_{transformer\;layers}$:

  6. t1 ← Apply layer normalization to the encoded patches.

  7. t2 ← Compute shifted window-based self-attention on t1 using a multi-head attention mechanism with projection dimensions.

  8. t3 ← Add t1 and attention outputs (Skip Connection 1).

  9. t4← Apply layer normalization to t3.

  10. t5← Pass t4 through a feed-forward MLP with predefined units and a dropout rate.

  11. t6← Add t3 and MLP outputs (Skip Connection 2).

  12. Update the encoded patches with t6.

 13: Final Feature Processing:

  •Apply final layer normalization to the encoded patches.

  •Flatten the feature representation.

  •Apply dropout regularization to the flattened features.

  •Pass the features through a fully connected classifier layer.

 14: Model Training:

  •Train the model using the AdamW optimizer with a learning rate of 5×$10^{-5}$.

  •Set the batch size to 16, meaning each iteration processes 16 images.

  •Define the window size as 7 × 7.

  •Compute loss using cross-entropy.

 15: 5-Fold Cross-Validation:

  •For each fold i from 1 to 5:

  21. Split the dataset into training and validation subsets.

  22. Train the ST on the training set.

  23. Evaluate on the validation set and compute classification metrics, including CA, F1, PR, RC, MCC and κ.

 26: Performance Evaluation:

  •Compute the mean performance metrics across all folds.

 27: Output: Class predictions and evaluation metrics.

 28: **end procedure**.

In this study, the Swin-Tiny variant, consisting of 2, 2, 6, and 2 transformer blocks at each hierarchical stage, was employed. The model’s effectiveness was ensured by meticulously tuning the hyperparameters. The learning rate was manually optimized to $5\times 10^{-5}$ after several trials to achieve the best performance. The batch size was set to 16, considering GPU memory limitations and to balance the model’s convergence process. The window size was configured to 7×7, while the patch size was defined as 4×4 to enhance feature extraction capabilities. These hyperparameters were carefully selected to optimize the model’s performance, particularly when working with high-resolution images. Moreover, the model was pre-trained on the ImageNet-1k dataset, which facilitated faster and more efficient learning. The number of layers to be frozen was determined through extensive trials, ensuring sufficient fine-tuning for the target task while preventing overfitting. The AdamW optimization algorithm was chosen due to its superior generalization ability, which was validated through numerous experiments. Its adaptive learning rate and weight decay properties have been shown to work effectively in similar DL tasks, making it an ideal choice. By incorporating local self-attention, a hierarchical design, and cross-window interactions, the ST has proven to be a powerful alternative to traditional CNNs and standard ViTs. As illustrated in Figs 5 and 6, these architectural innovations demonstrate the model’s ability to adapt to various computer vision applications, marking a significant advancement in transformer-based visual models. The fine-tuning process was carefully implemented to ensure the model’s high efficiency and performance.

### Classification stage and evaluation metrics

**Fig 7:**
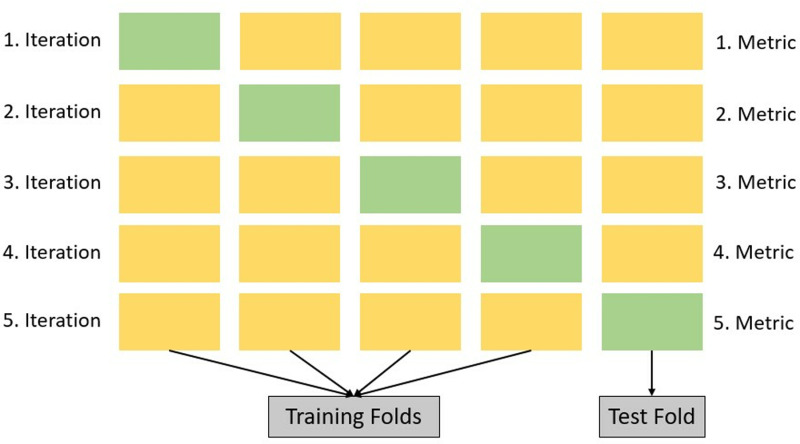
5.FCV procedure used for classification.

As illustrated in Fig 7, a 5-FCV strategy was employed to ensure the robustness and reliability of the classification model. In this approach, the dataset was divided into five equal-sized subsets. In each iteration, four folds were used for training while the remaining fold served as the test set [36]. This process was repeated five times, ensuring that each subset was utilized as a test fold exactly once. The final classification performance was determined by averaging the evaluation metrics across all five iterations, mitigating the risk of overfitting and providing a more generalized performance estimate.

To comprehensively assess the model’s classification capability, multiple evaluation metrics were computed, each offering distinct insights into its effectiveness [37]. The CA was determined as in Equation 4.


$$\begin{array}{c} CA=\frac{TP+TN}{TP+TN+FP+FN} \end{array}$$ (4)

where TP (True Positives) and TN (True Negatives) represent correctly classified instances of positive and negative classes, respectively, while FP (False Positives) and FN (False Negatives) denote misclassified instances. The PR and RC were also calculated to evaluate the reliability of the predictions as in Equations 5 and 6 [38].


$$\begin{array}{c} PR=\frac{TP}{TP+FP} \end{array}$$ (5)

$$\begin{array}{c} RC=\frac{TP}{TP+FN} \end{array}$$ (6)

Precision quantifies the proportion of correctly identified positive instances among all predicted positives, while recall measures the model’s ability to correctly identify all actual positive instances [38]. Furthermore, the F1, which balances PR and RC, was computed as in Equation 7 [38].


$$\begin{array}{c} F1\;=2\times \frac{PR\times RC}{PR+RC} \end{array}$$ (7)

The MCC is a powerful statistical metric used to evaluate classification performance, particularly in scenarios where class distributions are imbalanced. Unlike traditional accuracy measures, MCC considers all elements of the confusion matrix—TP, FP, TN and FN—providing a more holistic assessment of a model’s predictive capability [39]. The coefficient ranges from -1 to + 1, where +1 indicates perfect classification, 0 suggests no better performance than random chance, and -1 represents complete misclassification. The MCC is computed as in Equation 8. As formulated in Equation 8 [39], MCC effectively balances Type I and Type II errors, ensuring that classification performance is not skewed by class imbalance. Its ability to provide an interpretable correlation between predicted and actual classifications makes it a highly reliable metric for both binary and multi-class classification problems.


$$\begin{array}{c} MCC=\frac{\left(TP\times TN)-(FP\times FN\right)}{\sqrt{\left(TP+FP\right)}(TP\times FN)(TN\times FP)(TN\times FN)} \end{array}$$ (8)

Cohen’s Kappa is a statistical measure designed to quantify the agreement between two raters or classification systems while adjusting for the agreement that might occur by chance [40]. Unlike simple accuracy, which does not account for expected random agreement, κ provides a more rigorous assessment of classification reliability. The coefficient is defined as in Equation 9. where $\rho_{0}$ represents the observed agreement between classifications, and $\rho_{e}$ denotes the expected agreement based on random chance. The κ value ranges from -1 to + 1, where +1 signifies perfect agreement, 0 indicates agreement equivalent to random chance, and negative values suggest systematic disagreement. As expressed in Equation 9 [40], κ is particularly useful in multi-class classification, medical diagnoses, and annotation tasks, where ensuring classification reliability is crucial. By addressing the biases of raw accuracy, κ serves as a more insightful metric for evaluating classification consistency.


$$\begin{array}{c} \kappa =\frac{\rho_{0}-\rho_{e}}{1-\rho_{e}} \end{array}$$ (9)

This metric is particularly useful when dealing with imbalanced datasets, ensuring a fair evaluation of model performance. To gain deeper insights into classification effectiveness, the confusion matrix was constructed for each fold, visually representing the distribution of true and false classifications. Additionally, AUC values were generated to analyze the model’s discrimination capability. After completing all five iterations, the mean of each metric was computed using the following general formula as given in Equation 10.


$$\begin{array}{c} {Metric}_{avg}=\frac{1}{5}\sum_{j=1}^{5}{Metric}_{j} \end{array}$$ (10)

where ${Metric}_{j}$ represents the respective performance metric obtained in the $j^{th}$ iteration. This aggregated result provides a more stable and reliable estimation of the model’s classification performance, ensuring its applicability to unseen data.

## Results

This study introduces SwinFishNet, an advanced approach for automatic FSC that integrates the ST architecture with transfer learning. The proposed method was rigorously evaluated on three diverse datasets: the 12-class BD-Freshwater-Fish dataset, the 10-class SmallFishBD dataset, and the 20-class FishSpecies dataset. By leveraging DL and image processing techniques, this study achieves state-of-the-art performance in FSC. A detailed analysis was conducted using a 5-FCV strategy to ensure model robustness and generalizability. All images were resized to 224×224 pixels to meet the input requirements of the ST model. The Swin-Tiny variant was utilized, consisting of 2, 2, 6, and 2 transformer blocks across hierarchical stages. The model was trained with a learning rate of 5×10 ⁻ ⁵, a batch size of 16, and a window size of 7×7. A patch size of 4×4 was applied to facilitate efficient feature extraction and hierarchical representation learning. Performance was assessed using multiple evaluation metrics, including CA, F1, RC, PR, MCC and κ. The results, summarized in Table 5, highlighted the exceptional stability and performance of SwinFishNet across all datasets, underscoring its robust classification capabilities. The model demonstrated consistent high accuracy across all evaluation metrics, showcasing its adaptability and effectiveness in different FSC tasks. For the BD-Freshwater-Fish dataset, SwinFishNet consistently delivered strong results, with CA ranging from 0.9806 to 0.9875 across all folds. The model’s F1, PR, and RC remained well above 0.9700, demonstrating its reliability in distinguishing between species. Notably, the model achieved a remarkable MCC of 0.9862 in Fold 4, indicating its superior ability to manage class imbalances and correctly classify samples from all classes. The κ, reflecting the agreement between predicted and true labels, also remained highly throughout, further reinforcing the model’s effectiveness in real-world classification tasks.

**Table 5:** Performance metrics results for BD-Freshwater-Fish, SmallFishBD, and FishSpecies datasets using SwinFishNet for each fold.

Datasets	Fold	Metrics					
		CA	F1	RC	PR	MCC	κ
BD-Freshwater-Fish	Fold 1	0.9829	0.9856	0.9859	0.9856	0.9811	0.9811
	Fold 2	0.9806	0.9779	0.9796	0.9769	0.9786	0.9786
	Fold 3	0.9863	0.9792	0.9824	0.9773	0.9849	0.9848
	Fold 4	0.9875	0.9834	0.9852	0.9823	0.9862	0.9861
	Fold 5	0.9863	0.9874	0.9884	0.9867	0.9848	0.9848
SmallFishBD	Fold 1	0.9944	0.9943	0.9944	0.9944	0.9937	0.9937
	Fold 2	0.9978	0.9977	0.9971	0.9983	0.9975	0.9975
	Fold 3	0.9956	0.9963	0.9967	0.9960	0.9951	0.9951
	Fold 4	0.9980	0.9981	0.9980	0.9983	0.9978	0.9978
	Fold 5	0.9961	0.9962	0.9967	0.9956	0.9956	0.9956
FishSpecies	Fold 1	0.9956	0.9956	0.9939	0.9977	0.9953	0.9953
	Fold 2	0.9752	0.9706	0.9702	0.9778	0.9738	0.9736
	Fold 3	0.9983	0.9984	0.9981	0.9988	0.9982	0.9982
	Fold 4	0.9980	0.9981	0.9986	0.9976	0.9978	0.9978
	Fold 5	0.9987	0.9988	0.9986	0.9990	0.9986	0.9986

In the case of the SmallFishBD dataset, SwinFishNet achieved near-perfect classification results, with accuracy scores surpassing 0.9940 in all folds. The model’s performance remained consistent across different metrics, with F1, RC, and PR all reaching exceptional levels. The MCC, peaking at 0.9978 in Fold 4, demonstrated the model’s strong correlation between predicted and actual outcomes. These results underscored SwinFishNet’s capability to handle even subtle distinctions between different fish species, making it a powerful tool for fine-grained classification tasks in challenging datasets. For the FishSpecies dataset, SwinFishNet again demonstrated its versatility, with performance metrics across all folds exhibiting minimal variation. The accuracy remained above 0.9750 in all folds, with Fold 3 reaching a high of 0.9983. The model’s F1, PR, and RC were also consistently strong, reflecting its ability to generalize across diverse species. The MCC and κ for the FishSpecies dataset further highlighted the model’s precision and robustness, with Fold 5 achieving an outstanding MCC of 0.9986 and a κ of 0.9986. These results demonstrated that SwinFishNet excelled not only in large datasets but also in datasets with greater class complexity and variability.

**Fig 8:**
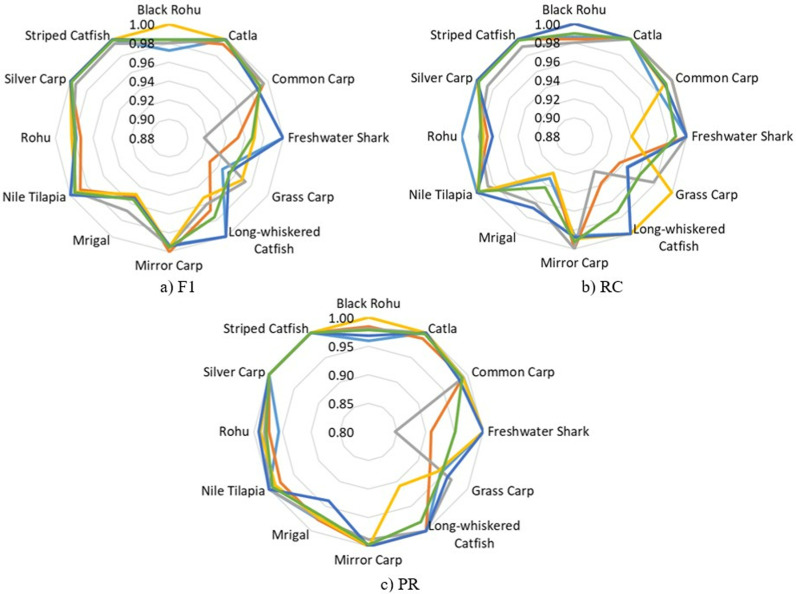
Class-wise F1, RC, and PR metrics for BD-Freshwater-Fish classification using SwinFishNet across 5-FCV.

**Fig 9:**
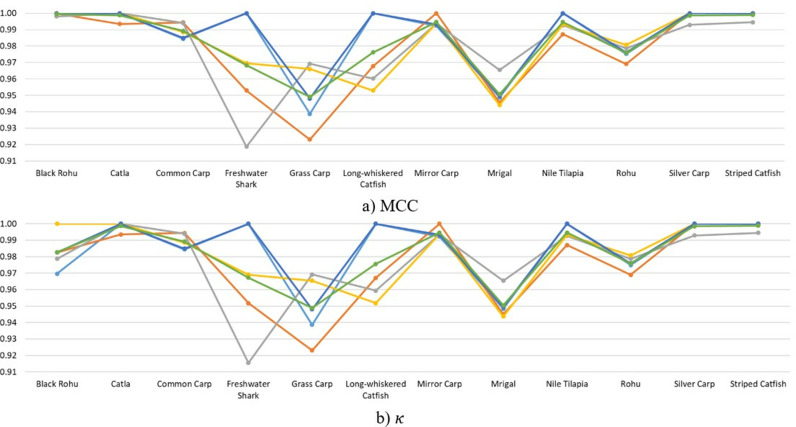
MCC and κ metrics per class for BD-Freshwater-fish classification using SwinFishNet with 5-FCV.

**Fig 10:**
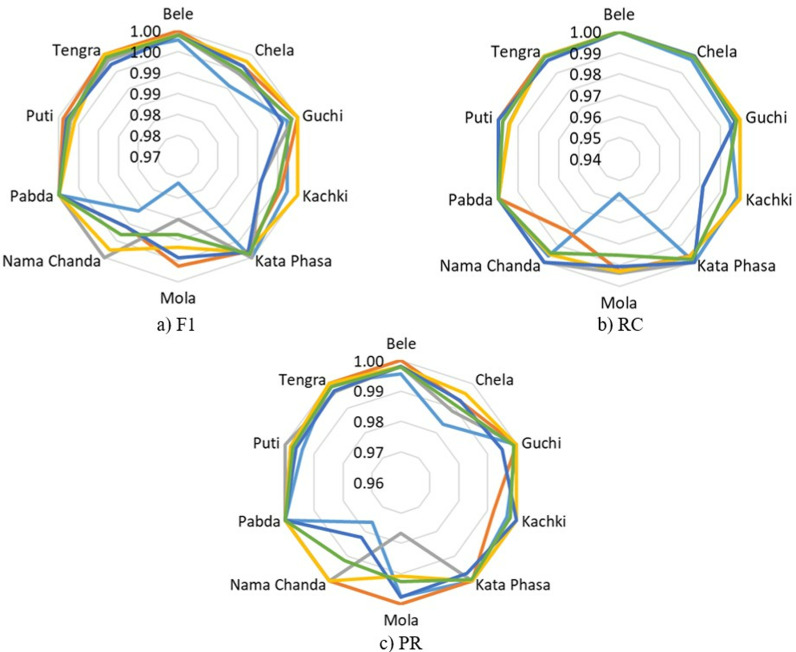
Class-wise F1, RC, and PR metrics for SmallFishBD classification using SwinFishNet across 5-FCV.

**Fig 11:**
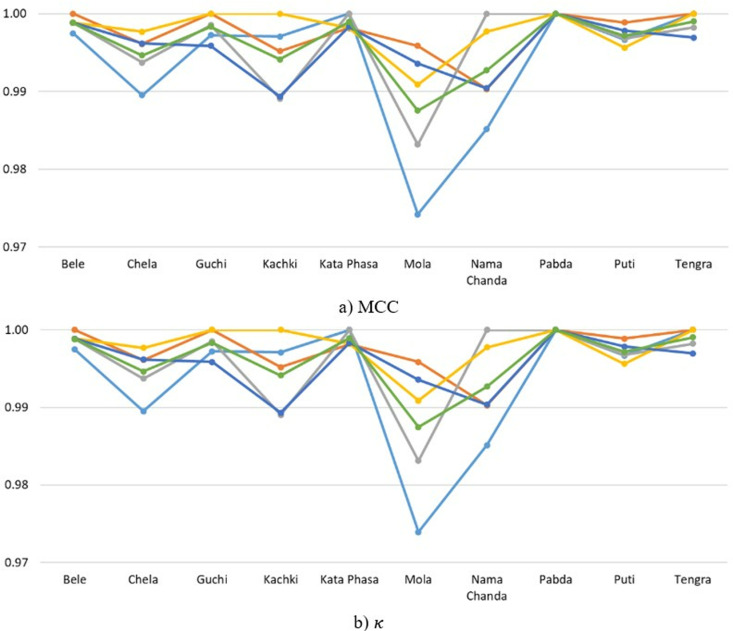
MCC and κ metrics per class for SmallFishBD classification using SwinFishNet with 5-FCV.

**Fig 12:**
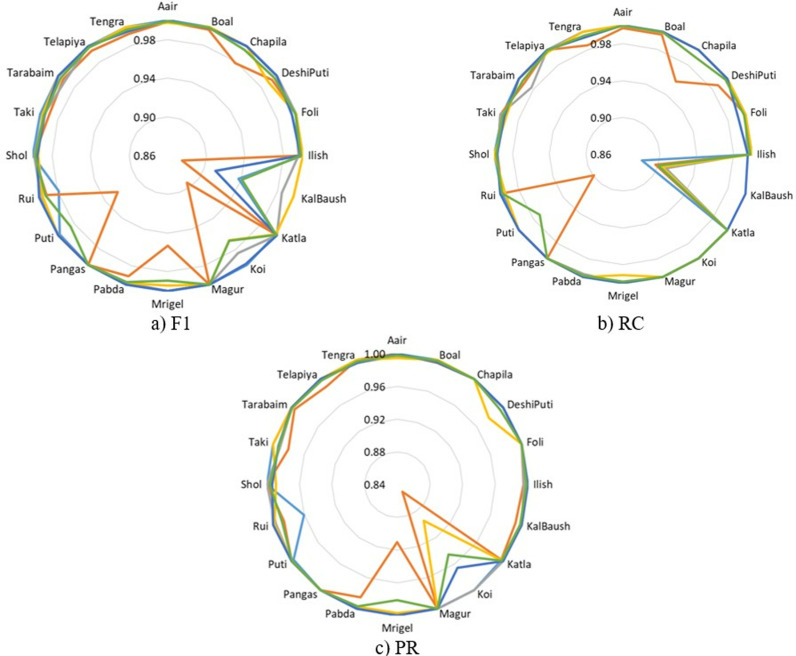
Class-wise F1, RC, and PR metrics for FishSpecies classification using SwinFishNet across 5-FCV.

**Fig 13:**
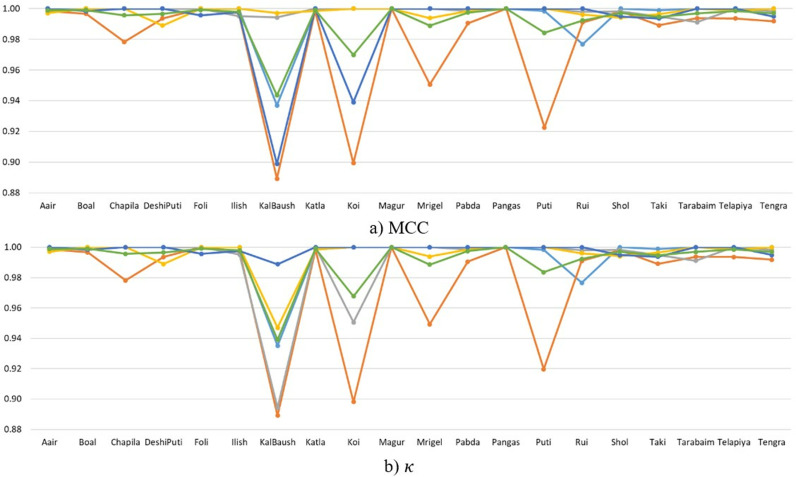
MCC and κ metrics per class for FishSpecies classification using SwinFishNet with 5-FCV.

The results presented in Fig 8 offer a comprehensive analysis of the SwinFishNet model’s performance across the BD-Freshwater-Fish dataset, evaluated over a 5-FCV. The class-wise F1, RC, and PR highlight the model’s remarkable robustness and exceptional discriminative power in classifying all twelve fish species. These results further emphasize the model’s consistent performance across the five folds, reinforcing its ability to deliver high CA even with varying data distributions. In terms of the F1, the model consistently achieved exceptional results, with average scores ranging from 0.9537 for Grass Carp to 0.9987 for Striped Catfish, confirming the model’s strong ability to distinguish between fish species. The SwinFishNet demonstrated impressive PR and RC across all species, further supporting its capacity to minimize FP and FN, crucial for applications where high classification reliability is required. Notably, for species such as Catla, Freshwater Shark, and Striped Catfish, the model maintained perfect PR and RC across all folds, indicating its ability to correctly classify these species under diverse conditions. The RC reflected a similar trend of consistent excellence, with an average RC of 0.9899 across all species. The model’s RC performance is particularly noteworthy for the Black Rohu, with values consistently close to 1 across all folds. This illustrates SwinFishNet’s effectiveness in identifying instances of this class, even in the presence of other closely related species. Additionally, the model’s average PR of 0.9782 across all species further demonstrates its capacity to achieve a balanced trade-off between FP and TP, a critical aspect in real-world FSC tasks where imbalanced data may otherwise lead to performance degradation. It was noted that all figures illustrated the classification performance across twelve fish species, with each fold distinctly represented by specific colors: light blue for Fold 1, orange for Fold 2, gray for Fold 3, yellow for Fold 4, dark blue for Fold 5, and green for the average across five folds. This color scheme was consistently applied in Figs 8–13 to ensure clarity and comparability of the results.

Furthermore, the classification performance of BD-Freshwater-Fish using SwinFishNet was comprehensively evaluated based on MCC and κ for each fish species through 5-FCV as given in Fig 9. The MCC ranged from 0.9188 to 1.0000 across different folds, with an average MCC exceeding 0.9946 for most species. Notably, Black Rohu, Catla, Rohu, Silver Carp, and Striped Catfish consistently achieved near-perfect scores, indicating that the model exhibited an exceptional ability to differentiate these species with minimal misclassification. The slightly lower MCC for Freshwater Shark and Grass Carp with 0.9682 and 0.9490, respectively, suggest that these classes had relatively higher intra-class variations or inter-class similarities, yet their classification remained highly accurate. Similarly, the κ followed a comparable trend, further validating the robustness of the model. The κ remained above 0.9155 across all classes, signifying a strong agreement between predicted and actual labels. The highest classification consistency was observed for Black Rohu, Catla, and Striped Catfish, with their κ converging towards 1.0000 in multiple folds. Freshwater Shark and Grass Carp exhibited the lowest κ with 0.9673 and 0.9489, respectively, yet their overall CA remained significantly high.

Moreover, Fig 10 presents the class-wise F1, RC, and PR, obtained for the SmallFishBD dataset using SwinFishNet with a 5-fold cross-validation strategy. The F1, which balanced both PR and RC, consistently remained above 0.9900 for most species, with the highest performance recorded for Kata Phasa, Pabda, and Tengra, all achieving an F1 of in multiple folds. Notably, even the species with relatively lower scores, such as Mola and Nama Chanda, attained F1 exceeding 0.9850, emphasizing the model’s reliability in handling challenging classifications. These results validate the robustness of SwinFishNet in accurately distinguishing fine-grained interspecies variations. The RC, measuring the model’s ability to identify positive instances, remained exceptionally high across all folds. Several species, including Bele, Chela, Guchi, Kata Phasa, Pabda, and Tengra, consistently reached perfect RC, demonstrating that the model effectively minimizes false negatives. Even for species with inherent variability, such as Mola and Nama Chanda, RC consistently exceeded 0.9800. Similarly, the PR, indicating the proportion of correctly classified samples, remained impressively high. Species like Kata Phasa and Pabda frequently achieved PR, affirming that the model effectively minimizes false positives. The lowest recorded PR, observed for Nama Chanda of 0.9822 in Fold 5, is still remarkably high, indicating that the classifier maintains superior specificity even in complex scenarios.

The proposed SwinFishNet model was comprehensively analyzed based on the MCC and κ using a 5-FCV approach on the SmallFishBD dataset, as illustrated in Fig 11. The obtained results demonstrate that the model exhibits exceptionally high CA across all fish species, achieving near-perfect classification performance for many of them. The consistently high MCC and κ, exceeding 0.9800, clearly indicate that the model effectively captures subtle morphological differences among species and remains robust against data imbalance. Notably, even for species such as Nama Chanda and Mola, which are relatively more challenging to distinguish, the MCC and κ never fall below 0.9800. This highlights the remarkable generalization capability of the model. These findings confirm that SwinFishNet provides a consistent and reliable classification performance across the entire dataset, rather than being limited to specific species.

In this study, the effectiveness of the proposed SwinFishNet method for FSC on a 20-class dataset was thoroughly evaluated. Specifically, class-wise F1, RC, and PR were computed through 5-FCV, and the results are presented in Fig 12. Additionally, the MCC and κ are shown in Fig 13. The class-wise F1 was consistently high, with many classes, including Aair, Boal, and Magur, highlighting the model’s precision in distinguishing between these species. The average F1 across all folds was 0.9988, reflecting the model’s robust classification performance. Similarly, the RC mirrored the F1, suggesting that the model was not only accurate in predicting positive instances but also sensitive enough to avoid false negatives. The RC for most classes was exceptionally high, especially for Aair, Boal, and Katla. PR also reinforced the model’s high classification capability. For most species, the PR remained strong, demonstrating the model’s ability to minimize false positives. Specific classes such as Puti, Magur, and Mrigel showed near-perfect PR, indicating the model’s capability to consistently identify these species with high accuracy. Moreover, the MCC and κ, as shown in Fig 13, corroborated the findings from the other metrics, emphasizing the model’s overall high performance. With average MCC and κ of 0.9969 and 0.9969, respectively, the results indicate a high level of agreement between the predicted and true class labels.

The confusion matrices provide a comprehensive visual representation of the model’s predictive performance, facilitating an in-depth analysis of CA while identifying both its strengths and limitations. Fig 11.a and b illustrate the average confusion matrices obtained through 5-FCV for the BD-Freshwater-Fish dataset with 12 classes, the SmallFishBD dataset with 10 classes, respectively. In these matrices, the horizontal axis represents the predicted classes, whereas the vertical axis corresponds to the actual classes. A thorough examination of these matrices offers valuable insights into the model’s overall accuracy as well as its ability to distinguish between specific categories.

**Fig 14:**
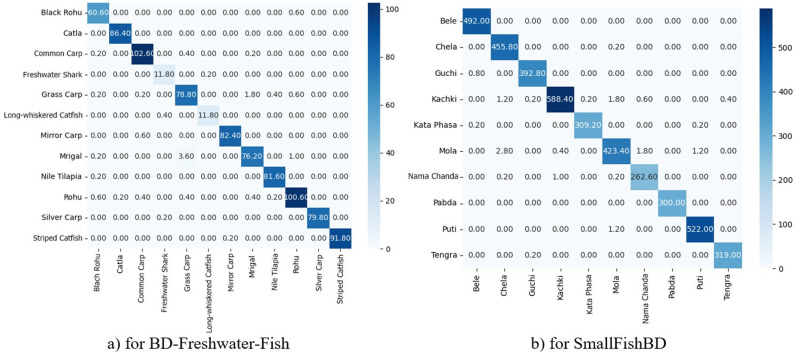
The average confusion matrices obtained through 5-FCV for the **a)** BD-Freshwater-Fish, **b)** SmallFishBD.

In the average confusion matrix presented in Fig 14.a for BD-Freshwater-Fish dataset, the model demonstrates strong CA for several species, such as Common Carp with 102.60 correctly classified instances, Rohu with 100.60, and Striped Catfish with 91.80, indicating its robustness in distinguishing these classes. However, minor misclassifications are observed in certain categories. For instance, Black Rohu exhibits a slight misclassification into Rohu with 0.60 instances, which suggests overlapping features between these species. Additionally, Freshwater Shark has a relatively lower recognition rate with 11.80 correctly classified instances, with minor misclassifications occurring across various species, highlighting potential challenges in differentiating this class due to limited distinguishing features or dataset imbalance. The results also indicated high precision for Mirror Carp with 82.40 and Nile Tilapia with 81.60, reinforcing the efficacy of the model in classifying these species. In the average confusion matrix presented in Fig 14.b for SmallFishBD dataset, the model demonstrates exceptional accuracy for most classes, with notably high correct classification rates for Bele with 492 correctly classified instances, Chela with 455.80, Kachki with 588.4, and Puti with 522.00. These results suggested that the model effectively differentiates these species, likely due to their distinct morphological characteristics. However, minor misclassifications are observed in certain cases. For instance, Guchi is occasionally misclassified as Bele with 0.8 instances, and Kachki shows slight confusion with Chela with 1.20 instances and Mola with 1.80 instances, indicating potential similarities in feature representations for these species. Similarly, Mola exhibits small-scale misclassification with Chela with 2.8 instances, suggesting that these species may share overlapping visual traits. Additionally, Kata Phasa with 309.20 correctly classified instances and Nama Chanda with 262.60 show slight misclassification rates, though their recognition remains reliable overall. Furthermore, the average confusion matrix for the FishSpecies dataset was computed to assess classification performance. The diagonal values, representing correctly classified instances, demonstrated high accuracy across multiple species. Specifically, the model correctly identified an average of 360.60 Aair, 330.00 Boal, 84.80 Chapila, 82.20 Deshi Puti, 112.20 Foli, 205.80 Ilish, 163.80 KalBaush, and 353.00 Katla. Additionally, it successfully classified 168.40 Koi, 114.80 Magur, 361.00 Mrigel, 352.40 Pabda, 186.80 Pandas, 302.80 Puti, 544.20 Rui, 284.20 Shol, 443.40 Taki, 251.20 Tarabaim, 411.40 Telapiya, and 285.00 Tengra.

To evaluate the effectiveness of SwinFishNet on imbalanced datasets, the AUC values were computed for three distinct datasets: BD-Freshwater-Fish, SmallFishBD, and FishSpecies. The results demonstrated consistently high classification performance across all datasets. For BD-Freshwater-Fish, the model achieved strong differentiation for Black Rohu with an AUC of 0.9995, Catla with 0.9997, and Common Carp with 0.9995. Similarly, Freshwater Shark and Grass Carp both exhibited AUC values of 0.9998, while Mirror Carp reached 0.9994. The model also performed well for Mrigal with 0.9981, Nile Tilapia with 1.0000, and Rohu with 0.9979. Additionally, Silver Carp showed an AUC of 0.9996, and Striped Catfish attained a perfect classification score of 1.0000. In the SmallFishBD dataset, the model maintained high accuracy across all species. Bele, Guchi, Kata Phasa, Pabda, and Tengra each recorded an AUC of 1.0000, while Chela and Nama Chanda achieved values close to perfect at 0.9999 and 0.9997, respectively. The species Kachki and Puti demonstrated strong classification performance with AUC values of 0.9998, whereas Mola recorded 0.9991, indicating a slight variation in classification confidence. For the FishSpecies dataset, the model showed exceptional classification capability, particularly for Aair, Boal, Foli, Katla, Magur, Pangas, Tarabaim, and Telapiya, all of which had AUC values of 1.0000. Chapila and Deshi Puti exhibited near-perfect classification with values of 0.9988 and 0.9998, respectively. KalBaush and Rui achieved 0.9983 and 0.9955, while Shol and Taki recorded 0.9985 and 0.9942. The lowest AUC value in this dataset was observed for Tengra at 0.9701, suggesting a minor challenge in distinguishing this species. These findings confirm the robustness of SwinFishNet in handling imbalanced datasets while maintaining high CA across diverse fish species. The consistently strong AUC values indicate that the model effectively distinguishes species with minimal misclassification, making it a reliable tool for fish species identification.

The comparative analysis of SwinFishNet against ResNet and EfficientNet, as presented in Table 6, underscores the superiority of the proposed model in FSC across three distinct datasets: BD-Freshwater-Fish, SmallFishBD, and FishSpecies. By evaluating CA, F1, RC, PR, MCC, and κ, it is evident that SwinFishNet consistently outperforms the other models, demonstrating its robustness in handling the complexities of FSC. For the BD-Freshwater-Fish, SwinFishNet achieved a CA of 0.9847, significantly surpassing ResNet with 0.9532 and EfficientNet with 0.9455. This improvement of approximately 3.10% over ResNet and 3.90% over EfficientNet highlights the ability of SwinFishNet to capture fine-grained visual features crucial for distinguishing similar fish species. The superiority extends to other performance metrics, with SwinFishNet attaining an F1 of 0.9827, exceeding ResNet with 0.9381 and EfficientNet with 0.9348, while also maintaining higher RC with 0.9843 and PR with 0.9817. The model’s MCC of 0.9831 and κ of 0.9831 further confirm its reliability in classification tasks. Similarly, in the SmallFishBD dataset, SwinFishNet achieved an exceptional CA of 0.9964, outperforming ResNet with 0.9707 and EfficientNet with 0.9874 by approximately 2.60% and 0.90%, respectively. This demonstrates the model’s effectiveness in classifying small-scale fish species, which often present challenges due to size variations and overlapping morphological traits. The F1 of 0.9965, RC of 0.9966, and PR of 0.9965 further reinforce the consistency and PR of SwinFishNet’s predictions, making it a highly reliable choice for small fish classification tasks. In the FishSpecies dataset, SwinFishNet maintained a high CA of 0.9932, outperforming ResNet with 0.9881 and EfficientNet with 0.9854. The 0.50% improvement over ResNet and 0.80% over EfficientNet may appear modest but is statistically significant in large-scale classification tasks, ensuring more precise identification across multiple fish categories. Additionally, the model’s F1 of 0.9923, RC of 0.9919, and PR of 0.9942 reaffirm its ability to handle imbalanced datasets while minimizing misclassification errors. The enhanced performance of SwinFishNet can be attributed to its transformer-based architecture, which enables superior spatial attention mechanisms, effectively capturing intricate patterns and subtle distinctions in fish morphology. Unlike ResNet and EfficientNet, which rely heavily on convolutional feature extraction, SwinFishNet leverages self-attention mechanisms, allowing for more adaptive and context-aware feature representations. This advantage is particularly beneficial in fish classification, where intra-class variations and inter-class similarities pose significant challenges.

**Table 6:** Average performance metrics across 5-FCV for each dataset for SwinFishNet, ResNet and EfficientNet.

Results	SwinFishNet			ResNet			EfficientNet		
	BD-Freshwater-Fish	Small FishBD	Fish Species	BD-Freshwater-Fish	Small FishBD	Fish Species	BD-Freshwater-Fish	Small FishBD	Fish Species
CA	**0.9847**	**0.9964**	**0.9932**	0.9532	0.9707	0.9881	0.9455	0.9874	0.9854
F1	0.9827	0.9965	0.9923	0.9381	0.9691	0.9888	0.9348	0.9875	0.9869
R	0.9843	0.9966	0.9919	0.9312	0.9689	0.9879	0.9434	0.9880	0.9869
P	0.9817	0.9965	0.9942	0.9532	0.9737	0.9901	0.9361	0.9874	0.9874
MCC	0.9831	0.9959	0.9928	0.9483	0.9675	0.9873	0.9403	0.9859	0.9845
κ	0.9831	0.9959	0.9927	0.9482	0.9672	0.9873	0.9396	0.9859	0.9845

## Conclusion

This study introduces SwinFishNet, an innovative model for FSC, harnessing the power of the ST’s hierarchical attention mechanism in combination with transfer learning techniques. The robust performance of the model was evaluated across three distinct datasets—BD-Freshwater-Fish, SmallFishBD, and FishSpecies—demonstrating its versatility in addressing the challenges posed by varying aquatic environments and class distributions. The consistently high CA, with average of 0.9847, 0.9964, and 0.9932 for the three datasets respectively, affirms the reliability and scalability of SwinFishNet in complex classification tasks.

A key strength of the ST is its ability to extract both global and local features, which proved to be crucial in distinguishing closely related species—something that conventional CNNs often struggle with. This capability not only enhances the precision of classification but also positions SwinFishNet as an advanced tool for various real-world applications that demand high accuracy in FSC. These include, but are not limited to, fisheries management, market quality control, and ecological monitoring. The model’s impressive performance across different datasets suggests its potential to adapt seamlessly to new and evolving classification tasks, particularly those requiring nuanced visual recognition. SwinFishNet’s architecture, driven by the transformer’s self-attention mechanisms, provides a significant advancement over traditional convolutional approaches. This makes it especially suited for tasks where subtle morphological differences between species must be identified. Moreover, the model’s adaptability to both balanced and imbalanced datasets further solidifies its utility in diverse ecological and environmental settings. The versatility demonstrated in this study makes SwinFishNet a promising candidate for real-time systems, offering the potential for integration into automated fisheries surveillance, species monitoring, and biodiversity assessments.

In terms of practical applications, the integration of SwinFishNet into real-world systems presents both exciting opportunities and challenges. While its performance is promising, scalability to real-time systems will require optimization, particularly in processing speed and resource efficiency. Real-world applications such as automated FSC in large-scale fisheries or marine conservation efforts may necessitate further model refinement to ensure rapid processing without sacrificing accuracy. Additionally, the model’s performance may vary under different environmental conditions and species representations, suggesting that future work will focus on expanding the model’s scope to incorporate more diverse data sources and environmental factors. Integration of multimodal data, such as water temperature, pH levels, and salinity, could further enhance the accuracy and robustness of the model in diverse ecological contexts.

This research not only underscores the transformative potential of transformer-based architectures in aquatic species identification but also highlights the need for continuous model improvement to address real-world complexities. In summary, SwinFishNet presents a robust, adaptable solution for automated FSC, with significant implications for marine biology, aquaculture, and environmental conservation. By refining and expanding this approach, we can further advance the study and management of aquatic ecosystems, offering valuable tools for ecological sustainability and biodiversity conservation.
